# Short-term outcomes of COVID-19 in pregnant women unvaccinated for SARS-CoV-2 in the first, second, and third trimesters: a retrospective study

**DOI:** 10.1590/1516-3180.2022.0323.R1.19082022

**Published:** 2022-10-28

**Authors:** Filiz Yarsilikal Guleroglu, Hatice Argun Atalmis, Icten Olgu Bafali, Gulser Bingol Dikdere, Irfan Dikdere, Murat Ekmez, Alpaslan Kaban, Fatma Karasabanoglu, Busra Seker Atas, Esra Selvi, Gulay Sumnu, Merve Topaktas, Merve Yasti Dayan, Sevilay Yavuz Dogu, Ali Cetin

**Affiliations:** I MD. Perinatologist, Department of Obstetrics and Gynecology, Haseki Training and Research Hospital affiliated with Health Sciences University, Sultangazi, Istanbul, Turkey.; II MD. Obstetrician and Gynecologist, Department of Obstetrics and Gynecology, Haseki Training and Research Hospital affiliated with Health Sciences University, Sultangazi, Istanbul, Turkey.; III MD. Obstetrician and Gynecologist, Department of Obstetrics and Gynecology, Haseki Training and Research Hospital affiliated with Health Sciences University, Sultangazi, Istanbul, Turkey.; IV MD. Obstetrician and Gynecologist, Department of Obstetrics and Gynecology, Haseki Training and Research Hospital affiliated with Health Sciences University, Sultangazi, Istanbul, Turkey.; V MD. Obstetrician and Gynecologist, Department of Obstetrics and Gynecology, Haseki Training and Research Hospital affiliated with Health Sciences University, Sultangazi, Istanbul, Turkey.; VI MD. Obstetrician and Gynecologist, Department of Obstetrics and Gynecology, Haseki Training and Research Hospital affiliated with Health Sciences University, Sultangazi, Istanbul, Turkey.; VII MD. Gynecologic Oncologist, Department of Obstetrics and Gynecology, Haseki Training and Research Hospital affiliated with Health Sciences University, Sultangazi, Istanbul, Turkey.; VIII MD. Obstetrician and Gynecologist, Department of Obstetrics and Gynecology, Haseki Training and Research Hospital affiliated with Health Sciences University, Sultangazi, Istanbul, Turkey.; IX MD. Obstetrician and Gynecologist, Department of Obstetrics and Gynecology, Haseki Training and Research Hospital affiliated with Health Sciences University, Sultangazi, Istanbul, Turkey.; X MD. Obstetrician and Gynecologist, Department of Obstetrics and Gynecology, Haseki Training and Research Hospital affiliated with Health Sciences University, Sultangazi, Istanbul, Turkey.; XI MD. Obstetrician and Gynecologist, Department of Obstetrics and Gynecology, Haseki Training and Research Hospital affiliated with Health Sciences University, Sultangazi, Istanbul, Turkey.; XII MD. Obstetrician and Gynecologist, Department of Obstetrics and Gynecology, Haseki Training and Research Hospital affiliated with Health Sciences University, Sultangazi, Istanbul, Turkey.; XIII MD. Obstetrician and Gynecologist, Department of Obstetrics and Gynecology, Haseki Training and Research Hospital affiliated with Health Sciences University, Sultangazi, Istanbul, Turkey.; XIV MD. Obstetrician and Gynecologist, Department of Obstetrics and Gynecology, Haseki Training and Research Hospital affiliated with Health Sciences University, Sultangazi, Istanbul, Turkey.; XV MD, PhD. Perinatologist, Department of Obstetrics and Gynecology, Haseki Training and Research Hospital affiliated with Health Sciences University, Sultangazi, Istanbul, Turkey.

**Keywords:** COVID-19, SARS-CoV-2, Pregnancy, Intensive care units, Intensive care units, neonatal, Outcome, Computed tomography, Intensive care unit, Neonatal intensive care unit

## Abstract

**BACKGROUND:**

Coronavirus disease 2019 (COVID-19) may be asymptomatic or symptomatic in pregnant women. Compared to non-pregnant reproductive-aged women, symptomatic individuals appear to have a higher risk of acquiring severe illness sequelae.

**OBJECTIVES:**

We assessed the clinical and laboratory characteristics and outcomes of pregnant COVID-19 patients unvaccinated for severe acute respiratory syndrome coronavirus 2 according to the trimester of pregnancy.

**DESIGN AND SETTING:**

This was a retrospective observational study conducted in a tertiary-level hospital in Turkey.

**METHODS:**

This retrospective study reviewed the clinical and laboratory characteristics and outcomes of 445 pregnant COVID-19 patients hospitalized during the first, second, and third trimesters of pregnancy and 149 other pregnant women as controls in a tertiary center from April 2020 to December 2021. All participants were unvaccinated.

**RESULTS:**

Overall, the study groups were comparable in terms of baseline clinical pregnancy characteristics. There was no clear difference among the study participants with COVID-19 in the first, second, and third trimesters of pregnancy. However, a considerably high number of clinical and laboratory findings revealed differences that were consistent with the inflammatory nature of the disease.

**CONCLUSIONS:**

The study results reveal the importance of careful follow-up of hospitalized cases as a necessary step by means of regular clinical and laboratory examinations in pregnant COVID-19 patients. With further studies, after implementing vaccination programs for COVID-19 in pregnant women, these data may help determine the impact of vaccination on the outcomes of pregnant COVID-19 patients.

## INTRODUCTION

Severe acute respiratory syndrome coronavirus 2 (SARS-CoV-2) is a ribonucleic acid virus that causes coronavirus disease 2019 (COVID-19).^1,2^ Within three months of the first case of COVID-19 being discovered at the end of 2019, the World Health Organization labeled the disease a pandemic.^3^ Although most COVID-19 patients present with mild to moderate symptoms,^4^ a small proportion of patients develop severe disease presentations, including respiratory failure, myocarditis, septic shock, and multiorgan failure.^5^ Acute respiratory distress syndrome develops in up to one-third of individuals hospitalized with severe pneumonia.^6^ Despite maximal cardiac support and invasive mechanical ventilation, mortality remains high in this population.^7,8^

Pregnant women comprise a distinct subgroup among those at increased risk of severe COVID-19 as these women are more sensitive to certain viral infections than the general population.^9^ The pathogenesis of SARS-CoV-2 infection in pregnancy is yet unknown.^10^ Affected pregnant women, like other COVID-19 patients, can be asymptomatic; however, they may also have several complications that worsen the outcome of their pregnancy. This is because during the course of their pregnancy, important pathophysiological deterioration can occur, including the development of increased pro-inflammatory states, cytokine production, and oxidative stress, all of which ultimately lead to cellular death.^11^ According to some research data, the severity of the illness in pregnant COVID-19 patients, is linked to perinatal risks and pregnancy outcomes.^12,13^ Pregnant women with suspected or confirmed COVID-19 should receive comprehensive obstetric management as well as mental health and psychosocial support to avoid complications^14^; in addition, they should be encouraged to receive regular perinatal care.^15^

For large population-based studies, there is a need to shed light on the clinical presentations developed under the effects of the baseline health status of patients to assess how SARS-CoV-2 infection during pregnancy affects pregnancy outcomes how it results in *in-utero* vertical transmission. In 2021, the implementation of COVID-19 vaccination in pregnant women was initiated in Turkey. Many hospitalized pregnant women among the general COVID-19 patients could not receive vaccination during the pandemic. We believe that a review of the clinical characteristics and outcomes of pregnant women treated for COVID-19 will be useful in improving the medical care of pregnant COVID-19 patients in the future, especially in the different trimesters of pregnancy.

## OBJECTIVE

This study aimed to assess the clinical and laboratory characteristics and outcomes of pregnant COVID-19 patients unvaccinated for SARS-CoV-2 during the first, second, and third trimesters of pregnancy.

## METHODS

This retrospective study was designed to compare the clinical and laboratory characteristics and outcomes of hospitalized pregnant COVID-19 patients, many of whom were referred to our tertiary care center. In addition, those without COVID-19 were used as controls. This study was conducted at a tertiary center, including 445 pregnant COVID-19 patients and 149 pregnant women as controls, from April 2020 to December 2021. The COVID-19 patients were divided into three subgroups according to the hospitalization in the first, second, and third trimesters. In addition, the findings from a cohort of pregnant women with uncomplicated, healthy pregnancies in the third trimester between 30–41 weeks and who delivered during the same period were used as control data for comparison. COVID-19 infection was confirmed by positive polymerase chain reaction for SARS-CoV-2 in nasopharyngeal samples, and all study participants were unvaccinated for COVID-19. This study was approved by the health authorities and the Ethics Committee for Human Research of our institution (Haseki Training and Research Hospital Ethics Committee of Clinical Studies; date: February 09, 2022; Registry No: 19-2022). During the study period, pregnant COVID-19 patients were managed following the national health guidelines, including oxygen administration, use of antiviral drugs, hydroxychloroquine, steroids, antibiotics, thromboprophylaxis, and bronchodilators. In addition, respiratory support was provided through mechanical ventilation after admission to the intensive care unit (ICU) when required.

Regarding the participants’ clinical characteristics, we collected the following data from the electronic patient records of our center: maternal age, gravidity, parity, ethnicity (native or Arabic), smoking status (yes or no), requirement of assisted reproductive technology for conception (yes or no), pre-pregnancy body mass index, mode of delivery (vaginal or cesarean), gestational age at the time of COVID-19 infection, gestational age at delivery, and maternal comorbidities (diabetes mellitus, hypertension, renal disease, asthma, and thyroid disease). In addition, in routine laboratory evaluation, hematological, biochemical, and inflammatory parameters were collected at admission, and maternal, fetal, and neonatal characteristics were recorded. A prophylactic dose of low molecular weight heparin was routinely prescribed for COVID-19 in our study population.

### Statistical analysis

Data are presented as means, standard deviations, medians with minimum and maximum values, and counts with percentage values. IBM SPSS for Windows v25 (IBM SPSS, Armonk, New York, United States) was used for the descriptive and comparative analyses. After the normality test, data were analyzed using analysis of variance (ANOVA) or the Kruskal–Wallis ANOVA test with Tukey’s or Mann–Whitney *U* tests, respectively. Next, categorical variables were analyzed using the chi-squared test. P values < 0.05 were considered statistically significant.

## RESULTS

This retrospective study included 445 pregnant COVID-19 patients and 149 healthy pregnant women as controls. Table 1 shows the clinical characteristics of pregnant women with and without COVID-19. Pregnant COVID-19 patients were divided into three groups according to the time of diagnosis in the first, second, and third trimesters. The rate of Arab immigrants in the control group was significantly higher than in the other groups (P < 0.05). In addition, the gestational age at delivery was significantly higher in the control group compared to the second-trimester participants (P < 0.05). There were no significant differences among the study groups regarding maternal age, gravidity, parity, pre-pregnancy body mass index, smoking status, use of assisted reproductive technology, mode of delivery, or maternal comorbidities (P > 0.05).

**Table 1.** Clinical characteristics of pregnant women with coronavirus disease 2019 (COVID-19) in the first, second, and third trimesters and of pregnant healthy controls

	Pregnant COVID-19 patients			Pregnant healthy controls	Significance
	First trimester (n = 45)	Second trimester (n = 120)	Third trimester (n = 280)	Thirdtrimester (n = 149)	
**Maternal age (years)**	29 (21–43)	30 (18–42)	29 (17–47)	28 (17–43)	NS
**Gravidity**	3 (1–9)	3 (1–10)	3 (1–9)	3 (1–12)	NS
**Parity**	1 (0–7)	1 (0–7)	1 (0–7)	2 (0–6)	NS
**Ethnicity, n (%)**					P = 0.001
Native	41 (91.1%)	111 (92.5%)	236 (84.3%)	106 (71.1%)	
Arabic	4 (8.9%)^a^	9 (7.5%)^a^	44 (15.7%)^a^	43 (28.9%)^b^	
**Smoking, n (%)**					NS
Yes	2 (4.4%)	2 (1.7%)	11 (3.9%)	2 (1.3%)	
No	43 (95.6%)	110 (98.3%)	269 (96.1%)	148 (98.7%)	
**ART pregnancy, n (%)**					NS
Yes	0 (0%)	4 (3.3%)	7 (2.5%)	2 (1.3%)	
No	45 (100%)	116 (96.7%)	273 (97.5%)	147 (98.7%)	
**Pre-pregnancy BMI (kg/m** ^2^ **)**	24.3 (20.1–26.2)	23.7 (21.3–27.4)	25.8 (22.4–37.3)	28.3 (19.9–45.7)	NS
**Mode of delivery, n (%)**					
Vaginal	22 (53.7%)	52 (44.4%)	109 (39.1%)	77 (52.7%)	NS
Cesarean	19 (46.3%)	65 (55.6%)	170 (60.9%)	69 (47.3%)	
**Gestational age at diagnosis (weeks)**	10 (6–14)	23 (15–28)	36 (29–42)		
**Gestational age at delivery (weeks)**	38 (34–40)^a,b^	38 (23–41)^b^	38 (30–42)^a,b^	38.5 (30–41)^a^	P = 0.001
**Maternal comorbidities, n (%)**					NS
DM	5 (11.1%)	5 (4.2%)	17 (6.1%)	9 (6%)	
HT	0 (0%)	3 (2.5%)	8 (2.9%)	11 (7.4%)	
Renal disease	0 (0%)	1 (0.8%)	2 (0.7%)	1 (0.7%)	
Asthma	2 (4.4%)	7 (5.8%)	10 (3.6%)	6 (4%)	
Thyroid disease	2 (4.4%)	12 (10%)	15 (5.4%)	7 (4.7%)	

ART = assisted reproductive technology; BMI = body mass index; DM = diabetes mellitus; HT = hypertension; NS = non-significant. Data are presented as medians with minimum and maximum values or as counts with percentages and were analyzed using the Kruskal–Wallis test followed by the Mann–Whitney *U* test for pairwise comparisons or the chi-square test as appropriate. Results of the pairwise comparisons were denoted with a letter (^a^ or ^b^). There was no significant difference between/among the study groups if they are marked with the same letter (P > 0.05), and there was a significant difference between/among the study groups if they are marked with different letters (P < 0.05).

Table 2 shows the laboratory parameters of pregnant women with and without COVID-19. The median lymphocyte, platelet count, and blood urea nitrogen values were significantly higher in the controls and in the first-trimester participants compared to the second- and third-trimester participants (P < 0.05). The median platelet counts were significantly lower in the first-, second-, and third-trimester participants than that in the controls (P < 0.05). The median values of aspartate aminotransferase were significantly higher in the first-, second-, and third-trimester participants than that in the controls (P < 0.05). The mean values of hemoglobin and hematocrit were significantly higher in the first-trimester participants than those in the other groups (P < 0.05). The median white blood cell count was significantly higher in the control group than those in the other groups (P < 0.05). The median white blood cell counts were significantly higher in the third-trimester participants than those of the first- and second-trimester participants (P < 0.05). The median neutrophil count was significantly higher in the control group than those in the other groups (P < 0.05). The median neutrophil counts were significantly higher in the second- and third-trimester participants than that in the first-trimester participants (P < 0.05). The median C-reactive protein level was significantly lower in the control group than those in the other groups (P < 0.05). The median C-reactive protein values were significantly lower in the first-trimester participants than those of the second- and third-trimester participants (P < 0.05). The median ferritin values were significantly higher in the first- and second-trimester participants than that of the third-trimester participants (P < 0.05). Compared to the values of the other groups, the median creatinine values were significantly lower in the second-trimester participants (P < 0.05). The median alanine aminotransferase value was significantly lower in the control group than those in the other groups (P < 0.05). The median alanine aminotransferase values were significantly lower in the third-trimester participants than those of the first- and second-trimester participants (P < 0.05). The median lactate dehydrogenase values were significantly higher in the controls and in the third-trimester participants than those of the first- and second-trimester participants (P < 0.05). The median D-dimer levels were significantly higher in the third-trimester participants than those of the first- and second-trimester participants (P < 0.05). There were no significant differences in the monocyte and fibrinogen levels among the study groups (P > 0.05). There were no significant differences among the first-, second-, and third-trimester participants regarding the values of direct, indirect, and total bilirubin, maternal pH, and bicarbonate (P > 0.05).

**Table 2.** Laboratory parameters of pregnant women with coronavirus disease 2019 (COVID-19) in the first, second, and third trimesters and of pregnant healthy controls

	Pregnant COVID-19 patients			Pregnant healthy controls	Significance
	First trimester (n = 45)	Second trimester (n = 120)	Third trimester (n = 280)	Thirdtrimester (n = 149)	
WBC (103 μL)	6.1 (2.6–22.3)^c^	7.5 (2.7–23.1)^c^	8.2 (3.2–20.8)^b^	10.7 (5.5–20.2)^a^	P = 0.001
NEU (103 μL)	4.1 (1.5–18.8)^c^	5.6 (0.9–21.6)^b^	6.3 (1.7–19.6)^b^	7.9 (3.7–20)^a^	P = 0.001
LYM (103 μL)	1.6 (0.3–3.1)^a^	1.2 (0.4–4.1)^b^	1.3 (0.2–3.8)^b^	1.8 (0.4–4.4)^a^	P = 0.001
MONO (103 μL)	0.4 (0.1–0.9)	0.4 (0.1–1.5)	0.4 (0.1–1.8)	0.5 (0.1–1.5)	NS
Hb (g/dL)	12.6 (8.8–14.8)^b^	11.1 (8.5–13.3)^a^	11.3 (5.6–15.6)^a^	11.6 (7–14.8)^a^	P = 0.001
Hct (%)	36.1 ± 3.4^b^	32.9 ± 3.1^a^	33.6 ± 3.8^a^	34.1 ± 3.9^a^	P = 0.001
PLT (103 μL)	207 (30–329)^b^	199 (67–412)^b^	197 (29–473)^b^	232 (110–441)^a^	P = 0.001
PCT (%)	0.2 (0.04–0.4)^a^	0.2 (0.07–0.38)^b^	0.2 (0.03–0.5)^b^	0.23 (0.09–0.41)^a^	P = 0.001
CRP (mg/L)	7.9 (0.3–159)^b^	31.7 (0.5–224)^c^	23.6 (0.7–197)^c^	3.4 (0.6–4.9)^a^	P = 0.001
Ferritin (ng/mL)	50 (10.7–778)^a^	50.9 (3.4–480)^a^	30.8 (3.5–900)^b^	–	P = 0.002
BUN (mg/dL)	15 (8–37)^a^	11.9 (5–77)^b^	11.3 (4.2–32)^b^	14 (9–31)^a^	P = 0.001
Creatinine (mg/dL)	0.46 (0.3–0.81)^a^	0.43 (0.25–2.38)^b^	0.46 (0.25–0.85^)a^	0.48 (0.34–0.72)^a^	P = 0.003
AST (IU/L)	24 (11–110)^b^	25 (8–169)^b^	22 (8–168)^b^	18 (12–35)^a^	P = 0.001
ALT (IU/L)	20.5 (8–145)^b^	16 (5–241)^b^	12 (4–222)^c^	11 (4–36)^a^	P = 0.001
Direct bilirubin (mg/dL)	0.1 (0.1–0.3)	0.2 (0.1–2.1)	0.2 (0.1–2.6)	–	NS
Indirect bilirubin (mg/dL)	0.2 ± 0.16	0.3 ± 0.16	0.3 ± 0.15	–	NS
Total bilirubin (mg/dL)	0.21 (0.1–0.7)	0.49 (0.2–2.1)	0.47 (0.2–2.7)	–	NS
LDH (IU/L)	190 (139–455)^b^	204 (100–679)^b^	236 (111–968)^a^	266 (244–481)^a^	P = 0.002
D-dimer (mg/L)	0.54 (0.3–33.9)^a^	1.1 (0.4–11.2)^a^	1.5 (0.3–36)^b^	–	P = 0.001
Fibrinogen (mg/dL)	410 (284–680)	437 (111–674)	446 (134–773)	559 (349–660)	NS
Maternal arterial pH	7.4 ± 0.1	7.4 ± 0.1	7.4 ± 0.1	–	NS
Bicarbonate (mmol/L)	24 ± 0.4	22.7 ± 2.8	21.7 ± 4.1	–	NS

WBC = white blood cell count; NEU = neutrophil; LYM = lymphocytes; MONO = monocytes; Hb = hemoglobin; Hct = hematocrit; PLT = platelet; PCT = platelet crit; CRP = C-reactive protein; BUN = blood urea nitrogen; AST = aspartate aminotransferase; ALT = alanine aminotransferase; LDH = lactate dehydrogenase; IU = international unit; NS = non-significant. Parametric data are presented as means with standard deviations and were analyzed using an analysis of variance (ANOVA) test followed by Tukey’s test for pairwise comparisons as appropriate. Non-parametric data are presented as medians with minimum and maximum values and were analyzed using the Kruskal–Wallis test followed by the Mann–Whitney *U* test for pairwise comparisons as appropriate. The results of the pairwise comparisons were denoted with letters (^a^, ^b^, or ^c^). There was no significant difference between/among the study groups if they are marked with the same letter (P > 0.05), and there was a significant difference between/among the study groups if they are marked with different letters (P < 0.05).

The clinical presentations of pregnant women with and without COVID-19 are presented in Table 3. The rates of intrahepatic cholestasis of pregnancy and intrauterine growth restriction were significantly lower in the controls and third-trimester participants than those in the first- and second-trimester participants (P < 0.05). The rate of preeclampsia was significantly lower in the control group than those in the other groups (P < 0.05). The rates of preterm birth and preterm prelabor rupture of membranes were significantly lower in the controls and first-trimester participants than those in the second- and third-trimester participants (P < 0.05). The rate of placental abruption was significantly higher in the third-trimester participants than those in the other groups (P < 0.05). The rates of oligohydramnios were significantly higher in the first- and second-trimester participants than those in the third-trimester participants (P < 0.05). The median length of hospitalization was significantly higher in the COVID-19 participants than that in the controls (P < 0.05). The median lengths of hospitalization were significantly higher in the second- and third-trimester participants than those in the first-trimester participants (P < 0.05). The proportion of second-trimester COVID-19 participants without drug use was significantly higher than those of the first- and third-trimester participants (P < 0.05). More second-trimester participants were using antiviral drugs compared to the first- and third-trimester participants (P < 0.05). The proportions of COVID-19 participants with no computed tomography imaging data and who presented with pneumonia findings in their computed tomography imaging were significantly higher in those in their second- and third-trimesters than in those in their first-trimester (P < 0.05). The proportions of the controls and COVID-19 participants in their third-trimester requiring admission to neonatal ICUs were significantly higher than those of the COVID-19 participants in their first- and second-trimesters (P < 0.05). There were no significant differences among the study groups in terms of stillbirth rates, birth weights, Apgar scores at 1 and 5 min, and cord blood pH values (P > 0.05). There were no significant differences among the pregnant COVID-19 patients in the first-, second-, and third-trimesters in terms of rates of disease severity, the need for oxygen, admission to the ICU, mortality, time from onset of symptoms to hospitalization, admission to the ICU, and length of stay in the ICU (P > 0.05). Among all pregnant COVID-19 patients, there was no significant difference in the severity of COVID-19 among pregnant women with comorbidities (P > 0.05).

**Table 3.** Maternal, fetal, and neonatal characteristics of pregnant women with coronavirus disease 2019 (COVID-19) in the first, second, and third trimesters and of pregnant healthy controls

	Pregnant COVID-19 patients			Pregnant healthy controls	Significance
	First trimester (n = 45)	Second trimester (n = 120)	Third trimester (n = 280)	Third trimester (n = 149)	
**Intrahepatic cholestasis of pregnancy, n (%)**	2 (4.4%)^b^	7 (5.8%)^b^	2 (0.7%)^a^	1 (0.7%)^a^	P = 0.003
**Preeclampsia, n (%)**	9 (20%)^b^	19 (15.8%)^b^	34 (12.1%)^b^	4 (2.7%)^a^	P = 0.001
**Preterm birth, n (%)**	4 (8.9%)^a^	23 (19.2%)^b^	45 (16.1%)^b^	13 (8.7%)^a^	P = 0.048
**PPROM, n (%)**	0 (0 %)^a^	11 (9.2%)^b^	16 (5.7%)^b^	2 (1.3%)^a^	P = 0.009
**Stillbirth, n (%)**	0 (0 %)	4 (3.3%)	4 (1.4%)	4 (2.7%)	NS
**Placental abruption, n (%)**	0 (0%)	4 (3.3%)^a^	19 (6.8%)^b^	2 (1.3%)^a^	P = 0.02
**Oligohydramnios, n (%)**	4 (8.9%)^a^	12 (10%)^a^	17 (6.1%)^b^	0 (0%)	P = 0.002
**IUGR, n (%)**	5 (11.1%)^b^	16 (13.3%)^b^	20 (7.1%)^a^	2 (1.3%)^a^	P = 0.002
**Length of hospitalization (days)**	4 (1–13)^c^	6 (1–45)^b^	5 (1–45)^b^	2 (1–8)^a^	P = 0.001
**Time from onset of symptoms to hospitalization (days)**	2 (1–10)	2 (1–10)	2 (1–10)	–	NS
**Disease severity, n (%)**				–	NS
Mild	33 (73.3%)	79 (65.8%)	207 (73.9%)		
Moderate	12 (26.7%)	30 (25%)	55 (19.6%)		
Severe	0 (0%)	10 (8.3%)	15 (5.3%)		
Critical	0 (0%)	1 (0.8%)	3 (1.2%)		
**Need for oxygen, n (%)**				–	NS
No	27 (60%)	51 (42.5%)	158 (56.4%)		
Non-invasive	18 (40%)	63 (52.5%)	111 (39.6%)		
Mechanical ventilation	0 (0%)	6 (5%)	11 (4%)		
**Treatment, n (%)**				–	P = 0.03
No drugs	28 (62.2%)^a^	49 (40.8%)^b^	158 (56%)^a^		
Steroids	3 (6.7%)	15 (12.5%)	28 (10.1%)		
Chloroquine	1 (2.2%)	4 (3.3%)	20 (7.2%)		
Antiviral drugs	10 (22.2%)^a^	40 (33.3%)^b^	58 (20.9%)^a^		
Steroids plus antiviral drugs	3 (6.7%)	12 (10%)	16 (5.8%)		
**CT imaging, n (%)**				–	P = 0.003
No CT imaging	44 (97.8%)^a^	103 (85.8%)^b^	212 (75.7%)^b^		
No diagnosis of pneumonia on CT	0 (0%)	1 (0.8%)	12 (4.2%)		
Diagnosis of pneumonia on CT	1 (2.2%)^a^	16 (13.3%)^b^	56 (20.1%)^b^		
**Admission to the ICU, n (%)**	0 (0%)	8 (6.7%)	18 (6.5%)	–	NS
**Admission to the ICU in terms of hospitalization (days)**	None	2 (1–3)	3 (1–8)	–	NS
**Length of stay in the ICU (days)**	None	6 (2–43)	6 (1–30)	–	NS
**Mortality, n (%)**	0 (0%)	2 (1.7%)	3 (1.1%)	–	NS
**Birth weight (g)**	2700 (2645–3030)	3110 (670–3950)	3121 (1578–4248)	3227 (1438–4410)	NS
**Apgar score**					NS
At 1 minute	9 (8–9)	9 (5–9)	9 (1–9)	9 (5–9)	
At 5 minutes	10 (9–10)	10 (8–10)	10 (3–10)	10 (6–10)	
**Cord blood pH**	7.4 (7.32–7.41)	7.32 (7.25–7.43)	7.35 (7.15–7.56)	7.35 (7.1–7.57)	NS
**Need for NICU admission, n (%)**	4 (8.9%)^a^	20 (16.7%)^a^	113 (40.4%)^b^	46 (30.9%)^b^	P = 0.001

PPROM = preterm prelabor rupture of membranes; IUGR = intrauterine growth restriction; CT = computed tomography; ICU = intensive care unit; NICU = neonatal intensive care unit; NS = non-significant. Data are presented as medians with minimum and maximum values or as counts with percentages and were analyzed using the Kruskal–Wallis test followed by the Mann–Whitney *U* test for pairwise comparisons or the chi-square test as appropriate. Results of the pairwise comparisons were denoted with a letter (^a^ or ^b^). There was no significant difference between/among the study groups if they are marked with the same letter (P > 0.05), and there was a significant difference between/among the study groups if they are marked with different letters (P < 0.05).

## DISCUSSION

This study examined the clinical and laboratory findings and outcomes of pregnant COVID-19 patients hospitalized in the first, second, and third trimesters. In addition, the clinical and laboratory parameters and maternal, fetal, and neonatal outcomes of the study groups were compared. The statistical analysis revealed no clear difference among the pregnant women who developed COVID-19 in the first, second, and third trimesters. However, some obstetric complications were seen specifically in some of the first-, second-, and third-trimester participants that were in accordance with the nature of obstetric complications, including intrahepatic cholestasis of pregnancy, preeclampsia, preterm birth, preterm prelabor rupture of membranes, placental abruption, oligohydramnios, and intrauterine growth restriction.

In a recent study evaluating the clinical and laboratory data of pregnant COVID-19 patients who did not have any comorbid conditions, some severe clinical symptoms were observed in the third-trimester patients. Additionally, the need for intensive care, the rates of cesarean section delivery, and the rates of preterm delivery were all elevated among pregnant women.^16^ In our study, participants with comorbidities were also included, and we found no significant difference among the pregnant COVID-19 patients in the first, second, and third trimesters in terms of rates of disease severity, admission to the ICU, and mode of delivery. However, in our study, the preterm birth rate was significantly higher in the first-trimester participants than in those diagnosed in the second and third trimesters.

Many studies have examined the laboratory characteristics of COVID-19 patients.^17,18,19^ Sun et al. analyzed the blood examination results between pregnant COVID-19 patients and non-COVID-19 pregnant women.^20^ They observed that pregnant COVID-19 patients had significantly fewer lymphocytes, significantly more neutrophils, and significantly higher C-reactive protein levels than controls. Chen et al. reported that 51 out of 116 pregnant women diagnosed with COVID-19 had lymphopenia,^21^ while Liu et al. reported that 12 out of 15 pregnant COVID-19 patients had decreased lymphocyte counts and 10 out of 15 had higher C-reactive protein levels.^22^ It has been suggested that pregnant COVID-19 patients’ blood parameters be closely monitored, and variations in these inflammatory indices have been linked to patient prognoses.^21^

Thrombocytopenia occurs in 5–40% of non-pregnant COVID-19 patients.^23^ The virus may directly infect bone marrow cells, or the immune system may aggregate and destroy platelets, thereby increasing platelet consumption via microthrombi production.^24^ In a study that included 21 COVID-19 patients in the second and third trimesters of pregnancy and 48 patients without COVID-19, those with COVID-19 had higher platelet counts and lower fibrinogen levels than those without COVID-19.^25^ This group also had lower platelet levels compared to the controls; however, there was no significant difference among the study groups regarding fibrinogen levels.

Several investigations have found that COVID-19 patients had abnormal aminotransferase levels.^26,27,28^ According to a cohort analysis of 1,099 COVID-19 cases, 21.3% had elevated alanine aminotransferase levels, and 22.2% had elevated aspartate aminotransferase levels.^17^ In our COVID-19 participants, elevated aspartate aminotransferase and alanine aminotransferase levels were also observed. This outcome could be attributed to the toxicity of the medications used during hospitalization as well as the clinical progression of the disease.

Numerous studies and meta-analyses have previously been published on the maternal and perinatal effects of the COVID-19 pandemic.^29,30^ Notably, our findings are consistent with previous research indicating that pregnant women that are positive for COVID-19 may experience worse maternal and neonatal outcomes.^31,32^ Simon et al. performed a cohort study to ascertain the effect of maternal COVID-19 on prematurity and obstetric outcomes.^33^ Their study highlighted that COVID-19 is associated with obstetric complications such as preeclampsia, diabetes mellitus, prematurity, and cesarean delivery. Although the results of our study are in accordance with those of the aforementioned study, the cesarean section rates did not increase in our population.

Tunc et al. evaluated the clinical and laboratory findings of pregnant COVID-19 patients in all trimesters of pregnancy. In their study population, the ICU admission rates of the study groups were in accordance with the findings of our study.^34^ We believe that managing hospitalized pregnant COVID-19 patients with a focus on monitoring clinical and laboratory findings and executing appropriate interventions could be a determining factor in reducing ICU admission rates.

Patients with mild-to-moderate SARS-CoV-2 infection, both symptomatic and asymptomatic, can recover within 7–14 days. According to the current statistics, the COVID-19 recovery rate differs across countries. Because all COVID-19 cases comprise both symptomatic and asymptomatic cases, with 90% of symptomatic patients having mild to moderate symptoms, it is estimated that more than 80% of all COVID-19 cases can be handled with minimal or no medical intervention. The effectiveness of inpatient or critical care, as well as the patient’s immune responses and baseline health status, determines recovery in the remaining cases.

The clinical circumstances of pregnant COVID-19 patients can rapidly deteriorate and result in respiratory failure. Therefore, meticulous follow-up of hospitalized cases is required, including monitoring of clinical and laboratory examination findings and performing therapeutic approaches. Changes in the cardiovascular system during pregnancy, such as increased heart rate, oxygen consumption, and decreased lung capacity, increase the risk of developing and progressing to severe acute respiratory distress syndrome. According to some studies, individuals may also exhibit signs and symptoms associated with effects on other organs.

Currently, no routine biomarkers for SARS-CoV-2 have been validated; however, several candidates are possible. The measurement of various general biomarkers in individuals with COVID-19 may aid in determining the severity of the disease and the efficacy of any treatments provided, particularly in patients with previous disorders linked to chronic conditions. Procalcitonin and C-reactive protein levels have been found to increase in various illness states, though not in all patients. Even if routine coagulation tests have low specificity for many infectious coagulation-related disturbances, they are not useless in the setting of COVID-19, especially when performing risk stratification in patients.

Taken together, the results of the relevant studies are in accordance with the findings of the current study. There are some contradictory results to those of previous studies, indicating the meaningful influence of pregnancy trimesters on the clinical presentations and outcomes of pregnant COVID-19 patients. The absence of a clear-cut difference among our study groups at the different trimesters of pregnancy may be due to the sample of our study, as it mainly consisted of COVID-19 participants who did not receive antenatal care from our center previously. In addition, the interpretation of our data requires caution, as there may be other undefined mechanisms linking COVID-19 to obstetrical outcomes in those with normal and pathologic conditions. There may be confounding factors associated with the range of gestational age in our study population, including confounding clinical factors such as maternal body mass index status, fetal sex, ethnicity, and the presence of undefined maternal infective and/or inflammatory states, as well as other systemic disorders. The inclusion of only healthy third-trimester pregnant women as controls may be considered a limiting factor from which to draw conclusions regarding the laboratory parameters of the current study. As a national strategy and part of the organization of the public health infrastructure, the assignment of COVID-19 patients to tertiary care hospitals, specifically the hospitalization of COVID-19 patients and compilation of experience in order to fight the challenges of severe complications, had a significant impact on reducing the morbidity and mortality and reducing the long-term sequelae of the condition.

## CONCLUSION

In conclusion, no clinical and laboratory parameters clearly demonstrated worse outcomes in this review of hospitalized and unvaccinated pregnant COVID-19 patients. Overall, the clinical and laboratory data from the current examination, in accordance with the piling results of clinical COVID-19 studies, support the importance of careful follow-up and management of hospitalized pregnant COVID-19 patients by experienced healthcare workers, including regular clinical and laboratory examinations. This review also supports the value of a management strategy conducted in accordance with the versatile involvement of several organ systems in order to discharge pregnant COVID-19 patients with acceptable outcomes similar to non-pregnant COVID-19 patients. With further studies following the implementation of vaccination programs for COVID-19 in pregnant women, these data may help determine the impact of vaccination on the outcomes of pregnant COVID-19 patients.
